# MRS Reveals Chronic Inflammation in T2w MRI-Negative Perilesional Cortex – A 6-Months Multimodal Imaging Follow-Up Study

**DOI:** 10.3389/fnins.2019.00863

**Published:** 2019-08-16

**Authors:** Amna Yasmin, Asla Pitkänen, Kimmo Jokivarsi, Pekka Poutiainen, Olli Gröhn, Riikka Immonen

**Affiliations:** ^1^A.I. Virtanen Institute for Molecular Sciences, University of Eastern Finland, Kuopio, Finland; ^2^Center of Diagnostic Imaging, Department of Cyclotron and Radiopharmacy, Kuopio University Hospital, Kuopio, Finland

**Keywords:** antioxidant, GPC+PCho, GSH, Ins, neurochemical profile, neuroinflammation, pericontusional zone

## Abstract

Sustained inflammation in the injured cortex is a promising therapeutic target for disease-modification after traumatic brain injury (TBI). However, its extent and dynamics of expansion are incompletely understood which challenges the timing and placement of therapeutics to lesioned area. Our aim was to characterize the evolution of chronic inflammation during lesion expansion in lateral fluid-percussion injury (FPI) rat model with focus on the MRI-negative perilesional cortex. T2-weighted MR imaging (T2w MRI) and localized magnetic resonance spectroscopy (MRS) were performed at 1, 3, and 6 months post-injury. End-point histology, including Nissl for neuronal death, GFAP for astrogliosis, and Prussian Blue for iron were used to assess perilesional histopathology. An additional animal cohort was imaged with a positron emission tomography (PET) using translocator protein 18 kDa (TSPO) radiotracer [^18^F]-FEPPA. T2w MRI assessed lesion growth and detected chronic inflammation along the lesion border while rest of the ipsilateral cortex was MRI-negative (MRI-). Instead, myo-inositol that is an inflammatory MRS marker for gliosis, glutathione for oxidative stress, and choline for membrane turnover were elevated throughout the 6-months follow-up in the MRI- perilesional cortex (all *p* < 0.05). MRS markers revealed chronically sustained inflammation across the ipsilateral cortex but did not indicate the upcoming lesion expansion. Instead, the rostral expansion of the cortical lesion was systematically preceded by a hyperintense band in T2w images months earlier. Histologic analysis of the hyperintensity indicated scattered astrocytes, incomplete glial scar, and intracellularly packed and free iron. Yet, the band was negative in [^18^F]-FEPPA-PET. [^18^F]-FEPPA also showed no cortical TSPO expression within the MRS voxel in MRI- perilesional cortex or anywhere along glial scar when assessed at 2 months post-injury. However, [^18^F]-FEPPA showed a robust signal increase, indicating reactive microgliosis in the ipsilateral thalamus at 2 months post-TBI. We present evidence that MRS reveals chronic posttraumatic inflammation in MRI-negative perilesional cortex. The mismatch in MRS, MRI, and PET measures may allow non-invasive endophenotyping of beneficial and detrimental inflammatory processes to aid targeting and timing of anti-inflammatory therapeutics.

## Introduction

The global incidence of traumatic brain injury (TBI) is estimated to be 69 million each year. In the United States 1.7–2.5 million people sustain a TBI annually and over 5.3 million are living with TBI-related disability [2005–2010 statistics; Centers for Disease Control and Prevention (CDC, 2017)]. The disability may emerge years after the injury, and apparently derives from slow detrimental pathologic processes and accumulated load of stressors – such as neuroinflammation – that could potentially be seized or alleviated by interventions, even after the acute post-injury phase (Pearn et al., 2017; Sun et al., 2017; Missault et al., 2018).

Lateral fluid-percussion injury (FPI) rat model for TBI is a clinically relevant well-characterized animal model of closed-head injury in humans (Thompson et al., 2005). The primary brain contusion site atrophies within the first weeks to months after the injury, and gradually forms a cerebrospinal fluid (CSF) -filled cavity upon debris clearance (Pierce et al., 1998; Immonen et al., 2009a). At chronic stage the “perilesional” or “pericontusional” cortex beyond the lesion edge and beyond the glial scar may appear normal in conventional T2w structural MRI. However, histologic analyses have shown widespread perilesional neuroinflammation (Chen et al., 2003; Ndode-Ekane et al., 2018), and fMRI has demonstrated functional defect (Niskanen et al., 2013). Moreover, perilesional cortex undergoes cellular and vascular degenerative as well as plastic repair processes which progress in parallel with local innate inflammatory response for months after injury (Hayward et al., 2011; Jullienne et al., 2016; Kyyriainen et al., 2017; van Vliet et al., 2017; Ndode-Ekane et al., 2018; Pitkanen et al., 2018).

Inflammation is postulated to play a crucial role in the long-term outcome of TBI, contributing to the development of posttraumatic epilepsy and increasing the risk of cognitive deficits and other morbidities (Burda et al., 2015; Corps et al., 2015; Hinson et al., 2015; Lucke-Wold et al., 2015; Ali et al., 2018; Kokiko-Cochran and Godbout, 2018; Missault et al., 2018). Various *in vivo* imaging approaches have been applied to assess the posttraumatic neuroinflammatory state. MRI studies utilizing intravascular contrast agents have detected chronically elevated blood-brain-barrier (BBB) permeability in the perilesional cortex (Li et al., 2016; Yoo et al., 2018) suggested to be associate with local inflammatory response (Dadas and Janigro, 2018). A proton MRS indicated an elevation in glial marker myo-inositol in the ipsilateral cortex acutely and during the first 2 weeks after controlled cortical impact injury (CCI) (Xu et al., 2011; Harris et al., 2012). PET and autoradiography studies utilizing translocator protein 18kDa (TSPO) radiotracers specific for reactive microglia and astroglia have demonstrated massive inflammation due to phagocytic microglia, peaking within the lesion on post-injury days 4–6. This is followed by a decaying signal from reactive astrogliosis at lesion edges, or microgliosis along injured axons, during the following weeks (Yu et al., 2010; Wang et al., 2014; Israel et al., 2016). However, the understanding of the evolution of chronic posttraumatic perilesional inflammation is far from complete.

We hypothesized that MRS profile of myo-inositol (Ins), glutathione (GSH), and total choline (GPC+PCho) will detect inflammation in MRI-negative cortex and that the increase or decrease of the concentration of the markers over time will differentiate whether the inflammation is sustained or attenuated. We refer to the combined information of glia marker Ins, oxidative stress marker GSH and membrane turnover marker GPC-PCho as *inflammatory MRS profile*. We further hypothesized that the inflammatory MRS profile will associate with the lesion expansion rate and the glial scar properties which are influenced by early and late immune responses (Sofroniew and Vinters, 2010). We assessed (a) lesion growth rate, (b) ^1^H-MRS neurochemical profile of the perilesional cortex at 1, 3, and 6 months after lateral FPI, (c) radiological inflammation characteristics around the lesion by T2w structural MRI, and (d) validated the imaging findings with the end-point histopathology. To complement the MRS and MRI findings, we collected PET data obtained with TSPO radiotracer [^18^F]-FEPPA in another animal cohort, and describe the chronic cortical TSPO expression along with structural MRI characterization.

## Materials and Methods

### Animals

Adult male Sprague Dawley rats (Harlan Netherlands B.V., Horst, Netherlands, *n* = 43, weight 387 ± 23 g at time of TBI) were used for the study. Rats were housed in individual cages under controlled conditions (temperature 22 ± 1°C, humidity 50–60%, 12 h light/12 h dark cycle) with *ad libitum* access to food pellets and water. All animal procedures were approved by the Animal Care and use Committee of the University of Eastern Finland and done in accordance with the guidelines of European Community Council Directives 2010/63/EU and 86/609/EEC. Animals were randomly grouped into TBI (*n* = 31) and sham-operated control (*n* = 8) groups. Four 8.5 months old naïve rats served as additional age matched non-operated control group for the 6 months post-operation data.

### Lateral Fluid-Percussion Injury

Traumatic brain injury was induced by lateral fluid-percussion described previously (McIntosh et al., 1989; Kharatishvili et al., 2006). Briefly, rats were anesthetized by injecting intraperitoneally (6 ml/kg) a cocktail of sodium pentobarbital (58 mg/kg), magnesium sulfate (127.2 mg/kg), propylene glycol (42.8%), and absolute ethanol (11.6%). A craniectomy of 5 mm diameter was drilled between bregma and lambda with a trephine on the left convexity (anterior edge 2.0 mm posterior to the bregma; lateral edge adjacent to the left lateral ridge) leaving the dura intact. Injury impact was induced by transient pressure fluid pulse of (21–23 m/s) by using fluid percussion device (AmScien Instruments, Richmond, VA, United States) against exposed dura. Impact pressure was adjusted to 3.5 atm in order to induce severe TBI. Pressure delivered ranged from 3.1 to 3.4 atm. Sham-operated controls received identical surgical procedure without impact.

### Magnetic Resonance Imaging (MRI)

Magnetic resonance imaging measurements were performed on 9.4 T horizontal magnet with linear volume transmit/quadrature surface receiver coil system (Bruker, ParaVision 5.1 system, RAPID coils). MRI scans were acquired at 1, 3, and 6 months from the day of injury or sham-operation. Anesthesia was induced with isoflurane (1.5%) in mixture of 70% N_2_O, 30% O_2_. Animals were secured to an animal holder with ear bars and bite bar. The holder was electronically heated to maintain a body temperature of 37°C throughout scanning. Respiratory rate was kept between 60 and 70 per min by adjusting isoflurane anesthesia level.

T2-weighted (T2w) coronal and horizontal multi-slice images were acquired with fast spin echo (TurboRARE) sequence with effective echo time 33 m/s, repetition time 2500 m/s, field of view 30 × 30 mm^2^ covered with 256 × 256 data points, RARE factor 8 (echo train length 8 echoes, shortest echo time 10.7 m/s, and echo spacing 11 m/s), 6 averages and scan time 8 min. Data set was composed of consecutive 17 coronal slices or 14 horizontal slices with thickness of 0.7 mm with no gaps. Cortical lesion volumes were obtained by intensity threshold based analysis of T2w images using in house written Matlab based analysis toolbox Aedes^1^. Two regions of interest (ROIs) were drawn in each animal: multi slice template of the ipsilateral cortex that mirrored the shape of the intact contralateral cortex, and a reference region in contralateral cortical gray matter. Pixels deviating >4 times the standard deviation (SD) from the contralateral cortical gray matter intensity were classified as abnormal tissue (i.e., the lesion). Lesion volume was then measured within the ROI template of intact, ipsilateral cortex. Subcortical atrophy was not included. Output is the “lost cortical volume.” The reproducibility in volumetric measurement was assessed by repeated (*N* = 5) volume measurements in the same animal with large cavity type lesion. Two times the SD (2 mm^3^ × 0.75 mm^3^) was then considered as an error margin for the volume measurement. Thus, the increase in volume >1.5 mm^3^ was considered as reliably detected growth in lesion volume.

In the rats undergoing the PET examination, 3D T1wt anatomical images were acquired with fast imaging with steady state precession (FISP) sequence with TR 8 m/s, TE 4 m/s, flip 15°, 175 μm^3^ resolution by FOV 40 mm × 40 mm × 40 mm and 228 × 228 × 228 matrix, 50 kHz spectral width and 3 averages (the contrast is a function of T1 and T2^∗^). FISP images were used as anatomical reference images for PET data.

### Magnetic Resonance Spectroscopy

Localized (single voxel) spectroscopy was performed in the perilesional cortex using PRESS sequence with echo time 11 m/s, repetition time 2500 m/s, bandwidth 4 kHz covered with 2048 points, and VAPOR (Tkac et al., 1999) water suppression. Fieldmap based shimming (MAPSHIM software package, Paravision 5.0, Bruker; Germany) was conducted to achieve water line width <18 Hz. MRS was acquired at 1, 3, and 6 months post-injury. Single voxel (1 mm × 3 mm × 5 mm) was placed in perilesional cortex avoiding CSF shown in Figure 4A, and 320 or 640 averages were collected. Reference water peak was acquired using the same pulse sequence without water supression. Figure 4B displays a representative spectrum, including the LCmodel (Provencher, 2001) fit and the residue. CSF volume fraction was quantified from T2w images as an overlap of cystic lesion or ventricle with the MRS voxel. All metabolite concentrations are shown as absolute concentrations with CSF-volume fraction correction. Also, the fact that reference water peak is higher due to CSF fraction within the voxel (tissue water content 80% while CSF water content 99%) is corrected for. As an alternative approach (results in Supplementary Data) concentrations were calculated relative to the total creatine (Cr+PCr; peak at 3.04). This is another way to account for the tissue atrophy, but it relies on the assumption that total creatine reflects the total cell number, and does not change as a disease effect. The spectra were analyzed with LC model and only results from metabolites with Cramér–Rao lower bound (SD%) <20 were included in further analysis. SD% were 10.5 ± 3.0%, 11.0 ± 3.3%, and 10.0 ± 2.1% for Ins (1, 3, and 6 months, respectively), 15.9 ± 3.8%, 17.3 ± 4.3%, and 14.9 ± 2.9% for GSH, and 10.3 ± 1.7%, 9.5 ± 1.9%, and 9.2 ± 1.8% for GPC+PCho, indicating that their concentrations could be reliably quantified with LCmodel fit.

### Positron Emission Tomography (PET) With [^18^F]-FEPPA

Small supplementary cohort of rats with lateral FPI (*n* = 3) was operated for [^18^F]-FEPPA-PET follow-up performed at 2 and 6 week (*n* = 1) or 4 and 8 week (*n* = 2) post-injury. Radiosynthesis of the TSPO radioligand [^18^F]-FEPPA was carried out as described by Wilson et al. (2008) with some modifications in the protocol.

Positron emission tomography scans were performed on Inveon DPET scanner (Siemens Medical Solutions, Knoxville, TN, United States). Anesthesia was induced with isoflurane (1.5%) in mixture of 70% N_2_O, 30% O_2_, rats were secured to an animal holder and the tail vein cannula was inserted. The holder was heated with warm water circulation to maintain a body temperature of 37°C. Respiratory rate was monitored and maintained between 55–70 by adjusting the anesthesia level. A 90 min dynamic PET was acquired after 28.8 ± 2.2 MBq [^18^F]-FEPPA tail-vein injection. Injection volume was 0.2–0.4 ml and it was given as 30 s injection. Frames of the last 40 min were summed for the maps and activity quantification. After PET acquisition the animal holder with the rat was inserted to adjacent CT scanner (Flex X-O, Gamma Medica-Ideas, Northridge, CA, United States) and CT images were acquired for structural reference. Reconstruction for the PET data was done using OSEM-2D algorithm (16 subsets and 4 iterations) after Fourier rebinning. Dead time, decay, scatter and attenuation corrections were applied. Co-registration between the 3D MRI, CT and PET images were done manually using Carimas 2.9 (Turku PET Centre, Finland). CT to MRI co-registration allowed overlay of PET on MRI. MRI images were acquired in previous or following week in respect to the PET scan. PET activity maps are presented as percent of injected dose per cubic centimeter (%ID/cc).

### Composite Neuroscore for Assessments of Somato-Motor Function

Composite neuroscore tests were done to all animals at baseline (before TBI), and on day 2, day 7, and day 14 post-TBI to assess injury severity and motor recovery. Briefly, animals were scored from 0 (severely impaired) to 4 (normal). Animals were assessed for forelimb flexion (2 indices) during suspension by tail, hindlimb flexion (2 indices) by lifting hind limbs up and back by tail of animal whilst forelimbs remained on hard surfaces, ability of animal to resist a lateral propulsion (2 indices) toward the left and right and angle board. A composite neuroscore (0–28 points) was generated by combining the scores for each of seven tests (Nissinen et al., 2017). To investigate if the recovery rate of sensory-motor functions could predict the type of the developing lesion or the inflammatory response detected by MRS, we calculated a measure for the degree of recovery in NS. The motor recovery was calculated as a difference in neuroscore on day 14 and day 2 which was normalized to the magnitude of impairment on day 2, that is [NS(day 14)-NS(day 2)]/[NS(baseline)-NS(day 2)].

### Tissue Processing and Histology

In the end of the 6-months follow-up, rats were perfused transcardially according to a paraformaldehyde fixation protocol described previously (Kharatishvili et al., 2007). Brains were removed and postfixed in 4% paraformaldehyde (PFA) for 4 h, cryoprotected in 20% glycerol in 0.02 M potassium phosphate buffer (pH7.4) for 48 h, frozen in dry ice and stored at −70°C. Brains were sectioned into coronal plane (1-in-10 series, 25 μm thick) on sliding microtome. First series of brain sections was collected in 10% formalin at room temperature and remaining series were stored in tissue collection solution (30% ethylene glycol, 25% glycerol in 0.05 M sodium phosphate buffer) at −20°C until further processing.

One series of sections was stained for thionin (Nissl) to analyze the cytoarchitecture of the lesioned cortex. An adjacent series of selected sections was stained for Perl’s Prussian blue to identify ferritic iron as described previously (Liu et al., 2014). Briefly, sections were incubated in freshly prepared solution of 5% potassium hexacyanoferrate trihydrate and 5% hydrochloric acid for 30 min followed by rinsing with water and counterstaining with nuclear fast red. To assess the glial scar characteristics and putative reactive astrogliosis in the perilesional cortex, selected sections were stained for glial fibrillary acidic protein (GFAP). Free-floating sections were immunohistochemically stained using monoclonal mouse anti-mouse GFAP antibody (#814369, Boehringer Mannheim, Germany). Briefly, the sections were treated with 1% hydrogen peroxide, washed in 0.02 M KPBS, pH 7.4, and blocked in 10% normal horse serum (NHS) and 0.5% TritonX-100 in KPBS. Sections were then incubated for 2 days (at +4°C) in primary antibody (1:2000) in 1% NHS and 0.5% TritonX-100 in KPBS. Thereafter, the sections were washed three times (2% NHS in KPBS), and incubated for 1 h in biotinylated anti-mouse IgG (1:200, #BA-2000, Vector Laboratories, Burlingame, CA, United States) diluted in 1% NHS, 0.3% TritonX-100 in KPBS. Sections were washed again and incubated for 45 min in avidin-biotin-peroxidase complex according to manufacturer’s instructions (Standard ABCkit, #PK-4000, Vector Laboratories). Then the incubations in the secondary antibody and in the avidin-biotin solution were repeated. The peroxidase activity was visualized with 0.05% 3,3′-diaminobenzidine (DAB, Pierce Chemicals, Rockford, IL, United States) solution containing 0.04%H_2_O_2_ and 0.02 M KPBS. The sections were mounted on gelatin-coated slides, dried overnight, and intensified with osmium tetroxide (OsO_4_, #191970, Electron Microscopy Sciences, Hatfield, PA, United States) and thiocarbohydrazide (#21900, Electron Microscopy Sciences, Hatfield, PA, United States) according to the method of Lewis et al. (1986). Finally, the slides were covered using DePex^®^.

### Statistics

Statistical analyses were performed using IBM SPSS Statistics (v.21). Differences in MRS neurochemical concentrations between TBI, sham and naïve groups were assessed with Kruskall-Wallis followed by a *post hoc* analysis using the Mann–Whitney *U*-test (applicable for non-normally distributed data). To assess the change over time in neurochemical concentrations between consecutive time points (1 vs. 3 months and 3 vs. 6 months) Wilcoxon’s test was performed and corrected for multiple comparisons (Bonferroni). Correlations between lesion volume and subsequent lesion growth, between apnea duration and lesion volume, between subacute Ins concentration and lesion volume, and between final GSH and GPC+PCh concentrations and preceding lesion growth were calculated using Pearson and corrected for multiple comparisons (Bonferroni). Data are reported as mean ± D. *P-*values < 0.05 were considered significant.

## Results

Acute post-impact mortality was 36% (11/31) in the TBI group and 0% (0/8) in sham-operated control group. Post-impact apnea was 31 ± 17 s with two rats with apnea time >50 s. Acute post-impact seizure-like behaviors (jerks) occurred in 48% (15/31) animals and their mean duration was 30 ± 9 s.

### Evolution of Cortical Lesion in T2w Structural MRI

**FIGURE 1:**
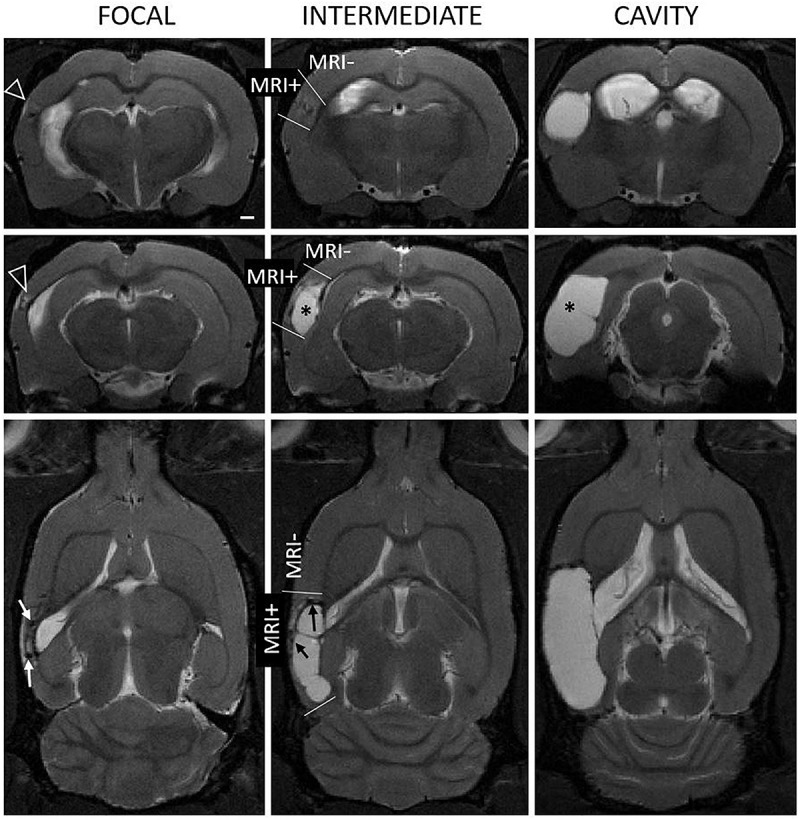
Heterogeneity of the endophenotype of cortical lesion in T2-weighted images at 1 month after lateral fluid-percussion induced TBI. Panel shows representative examples of “focal” **(left column)**, “intermediate” **(mid column)**, and “cavity-forming” **(right column)** endophenotypes, highlighting the 3D extent of the cortical lesions (see also Figure 3). **Top row:** Coronal views at the level of the rostral lesion tip. Note the rostral T2-enhancement (white triangle). **Middle row:** Center of the cortical lesion. **Bottom row:** Horizontal view depicting the rostro-caudal lesion extent. Contusion site is atrophied and the cavity filled with cerebrospinal fluid (asterisk). In focal and intermediate cases, the lesion was still expanding at 1 month and showed prominent T2w hyperintensity in the surrounding cortex, suggesting on-going inflammation (open arrowheads). We also found hyperintensity in association with hypointense hematomas or iron deposits (black and white arrows). MRI negative (MRI–) cortex refers to areas with normal gray matter contrast while MRI positive (MRI+) cortex refers to all observable abnormalities. Scale bar equals 1 mm.

**FIGURE 2:**
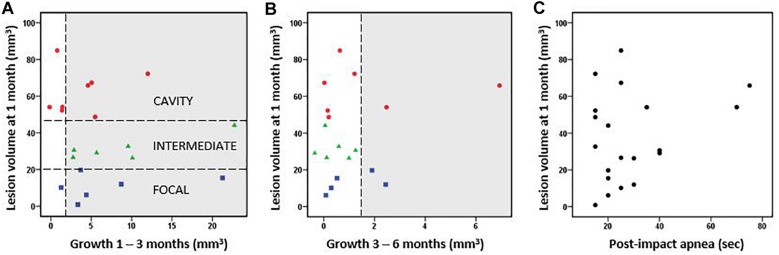
Cortical lesion volume and growth. **(A)** Rats were classified into three groups based on the cortical lesion volume at 1 month post-injury [horizontal lines in A separate the cavity (red, >48 mm^3^ lesion already at 1 month post-TBI), intermediate (green, 20–48 mm^3^), and focal (blue, <20 mm^3^) groups]. The lesion volume at 1 month did not correlate with the subsequent lesion growth from 1 to 3 months post-injury (Pearson, *p* > 0.05). **(B)** Lesion volume vs. lesion growth from 3 to 6 months post-injury. Thus, the growth of cortical lesion after the 1st post-injury month as calculated in cubic millimeters did not differ between the groups. Proportionally, however, the lesion volume two-folded in the focal group and increased almost 30% in the intermediate group between 1–6 months, whereas it stayed almost the same in the cavity groups. The growth >1.5 mm^3^ was considered as a true positive finding and highlighted in gray. The possible growth <1.5 mm^3^ is within the error margin of the quantification method. **(C)** Post-impact apnea duration did not correlate with the volume of cortical lesion (Pearson, *p* > 0.05).

**FIGURE 3:**
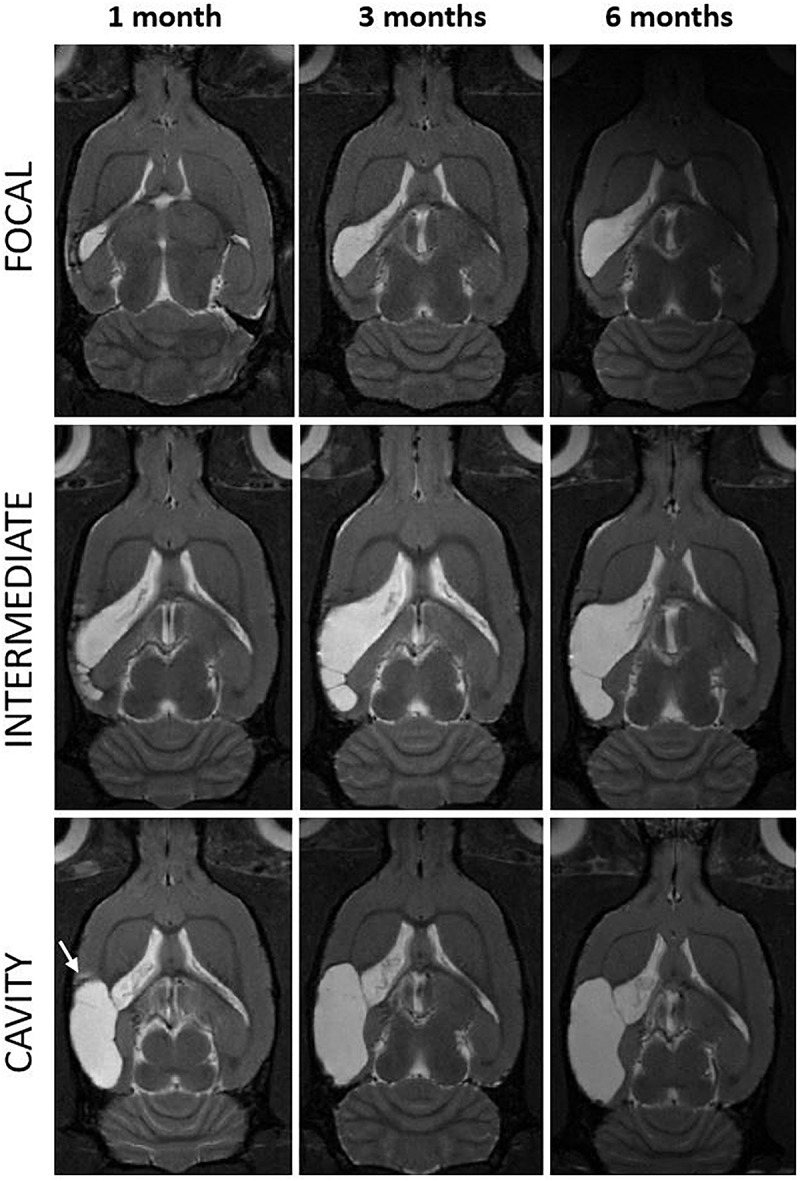
Progression of the cortical lesion from 1 to 6 months post-injury in focal, intermediate and cavity endophenotypes. T2-weighted horizontal images of three representative rats with lateral FPI **(rows)** over the 6-months follow-up **(columns)** are showing different rates of expansion of the cortical lesion. Lesion in a cavity case was already close to its maximal size by 1 month **(left and bottom)** while the focal and intermediate cases display slower rate of tissue atrophy and show trabecular lesions. They also show multisite hematomas and edema indicating on-going inflammation at 1 month. Note that also in the cavity case, at the rostral aspect of the cortical lesion there is MRI-detectable T2w hyperintensity at 1 month post-TBI (arrow). The MRI signs of inflammation are abolished by 3–6 months. Lesion has expanded over the areas that were abnormal in MRI at 1 month post-injury. In **(bottom right)** the cortical lesion cavity now covers also the area of the rostral T2w hyperintensity present at 1 month. Even though the rostral lesion growth in most of the cases was below the detection limit of the volumetric analysis (<1.5 mm^3^), it was apparent in visual analysis.

We measured the cortical lesion volume in T2w images encompassing all MRI positive (MRI+) cortical areas (Figure 1). Even in the hands of one experienced technician, the same impact force produced by the fluid-percussion device (3.27 ± 0.08 atm) resulted in a variable severity of cortical lesion within the TBI cohort large enough for detection of heterogeneity. Heterogeneity in early lesion growth rate is reflected by the lesion volume reached by 1 month post-injury (Figures 1, 2A). We classified the animals into three groups based on the lesion size at 1 month post-injury (Figure 2A). Consequently, 40% (8/20) of rats had developed a large (>48 mm^3^) CSF filled cystic lesion, extending throughout all cortical layers (“cavity”). 30% (6/20) of animals showed a small (<20 mm^3^) “focal” lesion. The remaining 30% (6/20) of rats had intermediate lesion volumes (26–45 mm^3^). Interestingly, the lesion had a heterogeneous, trabecular appearance, suggesting on-going necrosis and incomplete debris clearance. By 6 months, the “intermediate” cortical lesion endophenotype progressed to CSF-filled cavity. Figure 3 shows the lesion progression over time in representative cases of the “cavity,” “intermediate,” and “focal” endophenotypes. Multifocal hematomas in the contusion site were accompanied by hyperintense edema, and necrotic areas. This affected regions later atrophied into a lesion cavity. Narrow rim of cortical T2w-hyperintensity outside the primary lesion cavity indicative of local on-going inflammation was observable still 3 months post-injury. Rest of the ipsilateral cortex was MRI-negative (MRI-).

The subsequent growth of lesion cavity from 1 to 3 months, and thereafter, from 3 to 6 months post-injury varied greatly between the animals. The absolute volume of cortical tissue loss measured did not differ between groups. However, relative lesion volume loss between month 1 and month 3 was 6 ± 6% in the cavity, 26 ± 17% in the intermediate, and 115 ± 137% in the focal group. Thereafter, from 3 to 6 months the relative increase of the lesion volume was 2 ± 4% in the cavity, 1 ± 2% in the intermediate, and 5 ± 5% in the focal group. The lesion volume at 1 month did not correlate with the subsequent lesion growth (Figures 2A,B). The progression in lesion growth continued in 75% (15/20) of animals after 1 month. Of the 5 rats with no progression in lesion volume, 4 belonged into the cavity and 1 into the focal group. Between 3 and 6 months, the growth of lesion volume exceeded 1.5 mm^3^ (detection limit) only in 20% (4/20) of animals.

Since prolonged post-impact apnea may present a “second hit” and aggravate the aftermath of primary impact, we examined its association with the lesion volume. Apnea duration did not correlate with the lesion volume at 1 month post-injury (Figure 2C) or with lesion growth. However, the two cases with the longest apnea duration (>60 s) did develop large lesion cavities, and they showed a continuous chronic growth of lesion volume between 3 to 6 months (Figures 2B,C). Magnitude of impact pressure did not associate with the developing lesion volume.

### MRS Inflammatory Profile Stays Elevated From 1 to 6 Months in the MRI Negative Cortex

**FIGURE 4:**
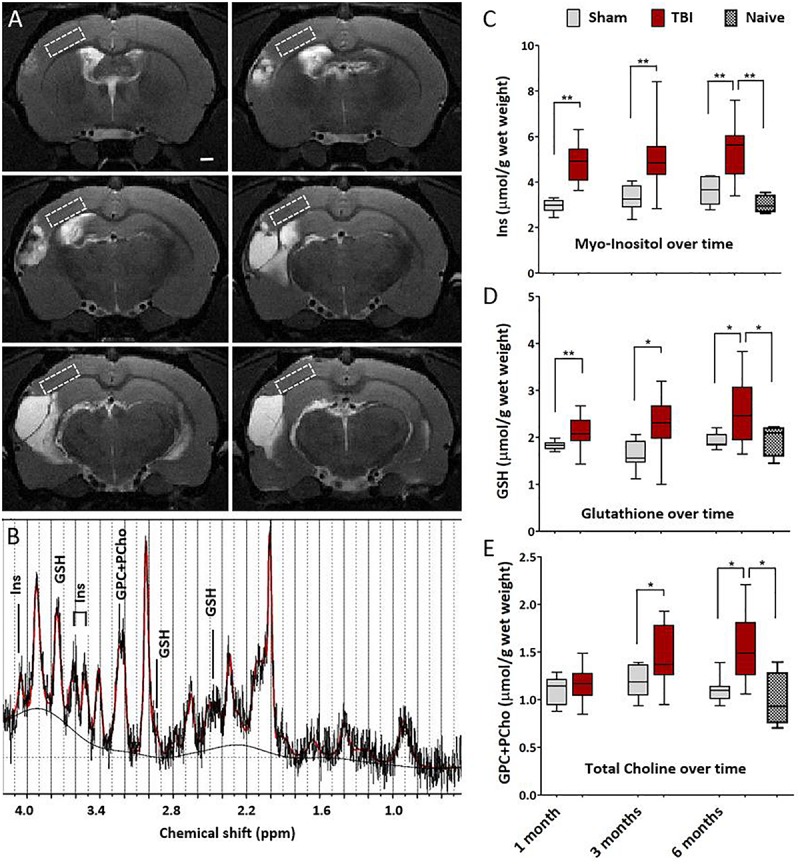
Magnetic resonance spectroscopy detects chronic cortical inflammation in MRI-negative perilesional cortex. **(A)** Location of the MRS voxel (white dashed square) at different rostro-caudal levels in the dorsal perilesional cortex in a representative animal at 6 months after TBI. Voxel was placed over the MRI- tissue in the T2w images. Note that at this chronic follow-up point the cortical thinning and progressive atrophy restricted the voxel size which had to be anticipated in the planning of the study. The strategy for voxel placement was to exclude the perilesional glial envelope and keep the distance from the lesion edge constant. In animals with the most extensive lesions and heavily enlarged ventricles, the overlap of the voxel with subcortical white matter (external capsule) and CSF-filled cavity could not be completely avoided, which was accounted for in the analysis. **(B)** Representative data showing a raw spectrum (black), LCmodel fit (red), and fitted baseline (solid black). The inflammatory markers myo-inositol (Ins, glial marker), glutathione (GSH, astrocyte-produced antioxidant) and glycerophosphocholine+phosphocholine (GPC+PCho, a marker of membrane turnover, both proliferation and degradation) are assigned to the corresponding peaks. LCmodel fit succeeded with Cramér-Rao lower bound SD% 10.5 ± 2.8 for Ins, 16.1 ± 3.8 for GSH, and 9.6 ± 1.8 for GPC+PCho. Absolute concentrations were corrected for the CSF volume fraction. Water reference is corrected for the 99% water content of CSF fraction while tissue water content was assumed to be 80%. **(C–E)** Box-plots showing the perilesional concentrations of Ins, GSH and GPC+PCho at 1, 3, and 6 months post-injury. The indicators for gliosis and oxidative stress were elevated through the 6 months follow-up. However, the increase in choline was not apparent untill at 1 month post-TBI, suggesting a more delayed tissue plasticity after the initial gliosis. Number of animals was 20 in the TBI, 8 in the sham-operated control, and 4 in the naïve group. Difference between the sham and TBI groups was assessed with Kruskall–Wallis with Mann–Whitney *post hoc* analysis; statistical significances: ^∗^*p* < 0.05 and ^∗∗^*p* < 0.01. No differences between the time points were found (Wilcoxon). Significances were corrected for multiple comparisons. The significances remained the same when calculated with the ANCOVA, including white matter volume fraction as covariate.

Magnetic resonance spectroscopy was performed to detect any subtle cortical inflammation. The MRS voxel was placed to MRI- perilesional cortex (voxel location depicted in Figure 4A). MRS revealed elevated concentrations of inflammation markers already at 1 month post-injury as compared to sham-operated experimental controls. Concentrations of glia marker Ins, antioxidant GSH and membrane turnover indicator GPC+PCho stayed elevated for 6 months after injury suggesting sustained inflammation (Figures 4B–E). Concentrations in shams 6 months post-operation did not differ from those in age matched naïve rats. Thus, unlike the T2w MRI, the spectroscopy could detect the chronic inflammation even at 6 months after injury.

### Association of the Dynamics of the Inflammatory MRS Profile to the Cortical Lesion Growth Rate

**FIGURE 5:**
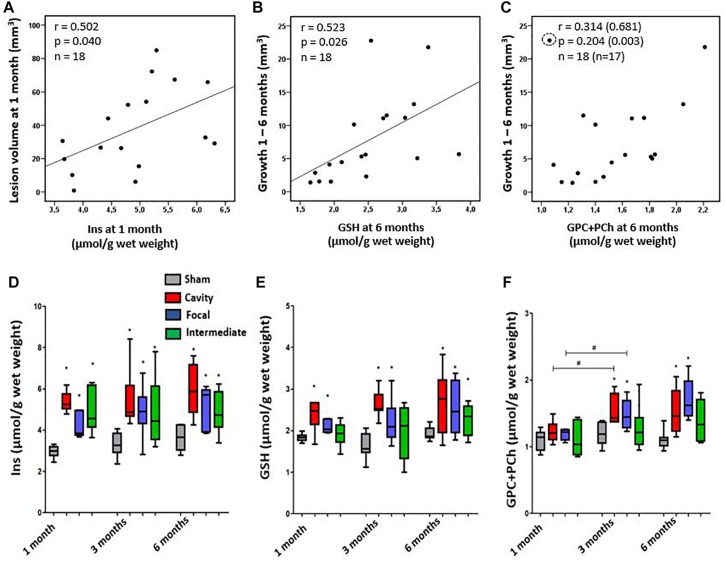
**(A–C)** Association of cortical inflammatory MRS profile with the structural endophenotype and lesion growth. **(A)** Myo-Inositol (Ins) concentration correlated with the lesion size at 1 month post-injury, but not at later time points. **(B)** Glutathione (GSH) concentration at 6 months post-injury correlated with the preceding growth of cortical lesion from 1 to 6 months. Similarly, **(C)** the total choline (GPC+PCho) concentration at 6 months was higher in animals with more pronounced preceding lesion growth, but correlation was not significant unless an outlier (circled) was excluded (values in brackets). Pearson correlations **(A–C)** are no longer statistically significant after Bonferroni correction for multiple comparisons (*p* > 0.05). **(D–F)** Dynamics of the inflammatory MRS profile over time in each structural endophenotype. **(D)** Endophenotypes did not significantly differ in their Ins dynamics, although the group with focal lesion had the lowest Ins level at 1 month and showed a trend toward increased Ins levels thereafter. **(E)** Antioxidant glutathione (GSH) produced by astroglia was elevated in all endophenotypes, but it was the highest in the cavity forming group. Temporal dynamics of the MRS markers did not differ between the groups. **(F)** The level of total choline (GPC+PCho) did not differ from controls at 1 month post-TBI, but became elevated thereafter, indicating comparable membrane turnover in both the cavity forming and focal lesion groups. Statistical significances: ^∗^*p* < 0.05 and (Mann–Whitney *U*-test between the groups); #*p* < 0.05 (Wilcoxon test, comparison of 1 and 3 months data from the same rats).

Next, we investigated how the inflammatory MRS profile reflected the early lesion growth until 1 month and the subsequent chronic lesion growth between 1–6 months. Findings are summarized in Figures 5A–C. Volume of the cortical lesion at 1 month associated with the 1-month Ins levels, suggesting that the early growth rate relates to the amount of glia in MRI- cortex. The MRS markers analyzed at 1 month post-injury did not predict the subsequent growth of cortical lesion. However, the levels of MRS markers at the chronic 6-month time point associated with the magnitude of the preceding lesion growth. GSH and GPC+PCho concentrations were the highest in animals with the greatest lesion growth during the preceding 1 to 6 months.

We found a remarkable variation in the dynamics of MRS markers in individual animals during the follow-up. Particularly, the two extremities were conspicuous: animals with the Ins, GSH and GPC+PCho concentrations progressively increasing or remaining at a high level, and animals with the levels of MRS markers decreasing over time. Therefore, we assessed whether the specific patterns in MRS markers would associate with the structural endophenotype of the cortical lesion and the lesion growth rate. Figures 5D–F shows the dynamics of different MRS inflammatory markers in different structural endophenotypes. In the cavity forming cases the levels of perilesional Ins between 1–3 months post-TBI remained rather stable. In the animals with focal lesions the direction of change in absolute Ins levels varied (Figure 5D). When the level of Ins were normalized to total creatine (Supplementary Figure 1), they decreased over time in the cavity group, indicating attenuating of the inflammation (Supplementary Figure 1A). Antioxidant GSH was the highest in the cavity forming cases, but its concentration or dynamics did not differ from that of the focal endophenotype. Total choline, GPC+PCho, a marker of membrane turnover, showed a late increase, starting at 3 months, and remained elevated at 6 months. It did not show endophenotype-specific dynamics. Thus, the dynamics of individual compounds Ins, GSH or GPC+PCho could not be linked with the structural endophenotype.

Unsupervised hierarchical clustering was performed to test if the three perilesional MRS markers *as a combination* would cluster the three endophenotypes of cortical pathology correctly within the TBI group. While the sham-operated experimental controls clustered separate from the animals with TBI based on the levels of Ins, GSH and GPC+PCho, the clustering did not separate the TBI endophenotypes correctly. Supplementary Figures 2A,B shows the 3D clustering and the best of the dendrograms at 1, 3, and 6 months post-TBI. Taken together, the inflammatory MRS profile (Ins, GSH and GPC+PCho together) did not associate with the growth rate of the cortical lesion.

### Somatomotor Impairment and Spontaneous Recovery From Day 2 to Day 14 Post-TBI Did Not Predict the Endophenotype of Cortical Pathology at 1 Month

Our data suggested that the rate of the progression in cortical lesion volume reports on the capacity of brain immunoresponse in a given rat. We then assessed whether progression of post-TBI somato-motor function (i.e., spontaneous recovery) would be linked to the evolution of the endophenotype of cortical lesion. Recovery of post-TBI performance in neuroscore did not differ between the structural endophenotypes (Supplementary Figure 2C), neither did the severity of drop in the neuroscore from baseline to 2 day post-injury. Neuroscore results did not correlate with the developing lesion size or growth. Thus, neuroscore did not predict the lesion extent, its progression, or endophenotype. Neither did the neuroscore recovery index show any correlations with MRS inflammation markers. The impact pressure in FPI induction did not correlate with neuroscore results.

### Association of the Glia Scar Properties With the Lesion Expansion and With the Level of MRS Inflammatory Markers in MRI- Cortex

Next, we studied the astroglia scar outlining and encapsulating the lesion. We hypothesized that the glia scar properties would differ in animals with different rate of lesion expansion. We also hypothesized that animals with leaky or incomplete glia scar would have higher levels of MRS inflammatory markers in MRI- parenchyma.

### Glia Scar Histopathology at 7 Months Post-injury Showed Non-mature and Diffuse Glia Barrier at Site of On-Going Lesion Expansion During Preceding 3 to 6 Months

**FIGURE 6:**
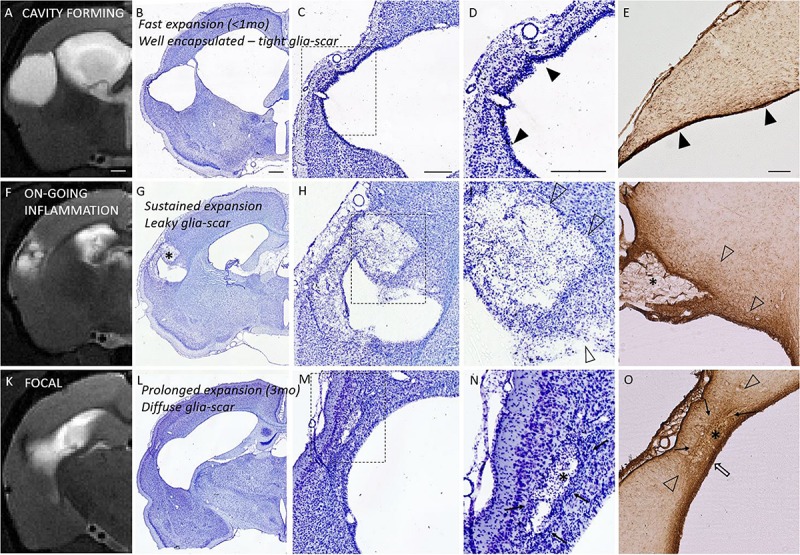
Glia envelope maturation stages and their association with the structural endophenotypes. Panel shows three example animals representative of mature (*top row*), leaky (*middle row*), and diffuse (*bottom row*) glia scar around the lesion. T2-weighted MRI images (*first column*) are shown in parallel with the corresponding Nissl stained sections (2nd column and magnified views in 3rd and 4th column) as well as GFAP stained sections (last column). *Top row* shows tight narrow glia scar (solid arrowheads in panels **D,E**) completely encapsulating the lesion. This mature glia envelope is observed particularly in cavity forming endophenotype, in which cavity develops fast (within first weeks after injury, <1 month). *Middle row* shows a case, where glia scar is incomplete and leaky with dispersed gliosis (open arrowheads in panels **I,J**), there is on-going inflammation, and the lesion is showing sustained expansion in MRI. *Bottom row* shows focal endophenotype with lesion and all its’ pockets surrounded by gliosis. While the glia encapsulates the lesions, the scar is not tight and narrow but a thick diffuse one, putatively still maturating (cloud of glia is indicated by solid arrows; thicker glia envelope indicated by open arrow). Iron residues are found at lesion edges in all animals: embedded into the tight glia envelope and aggregated and/or dispersed in the pockets (asterisks in **G,J,N,O**) with different degree of glia shielding. T2w images are taken 6 months post-injury and the tissue is harvested 7 months post-injury. While on-going inflammatory T2w-MRI signature **(F)** is rare 6 months post-injury, it is observed in 75% of the TBI animals 1 month after injury. It manifests in the rostral tip of the lesion in cavity endophenotype, and more widely around the lesion in intermediate and focal endophenotypes. Nissl staining of the cytoarchitecture of the cortical lesion is shown with two magnifications (**D,I,N** are magnifications of the dashed square in panels **C,H,M**, respectively). Scale bars equal 1 mm (MRI; **A,F,K**), 1 mm (Nissl of whole hemisphere; **B,G,L**), 500 μm **(C,H,M)**, 500 μm **(D,I,N)**, and 200 μm (GFAP; **E,J,O**).

Properties of the glia envelope were examined in the endpoint histology. A dense, tight and thin glial layer was assigned as a “mature” glial scar. A dispersed and diffuse gliosis was considered as a “maturing or immature” glial scar. Figure 6 shows the association between the Nissl cytoarchitecture at the lesion edge, GFAP characteristics and the structural MRI endophenotypes. Based on the GFAP and Nissl stainings all rats in the cavity group had a mature, narrow glial scar outlining all 3D aspects of the cortical lesion. The only exception was the rostral tip of the lesion, in which the scar remained diffuse. The animals in the focal group showed a thick cloud of GFAP+glia surrounding the lesion as well as the accompanying iron residues. Thus, even though the perilesional glial barrier was present in the focal group, it appeared diffuse and differed from the tightly packed mature envelope in the cavity group. All focal cases had dispersed gliosis rostral to the lesion.

Iron staining (Perl’s Prussian Blue) showed both diffuse free iron and phagocyted intracellular iron at the areas of dispersed gliosis. Iron and iron aggregates were found embedded into the tightly packed perilesional mature glial scar as well as in glia-encapsulated isolated pockets. Histologically detected iron was also detected by the T2w MRI (Figure 6).

### T2-Hyperintense Band in MRI Next to the Glia Scar Preceded the Subsequent Lesion Growth

**FIGURE 7:**
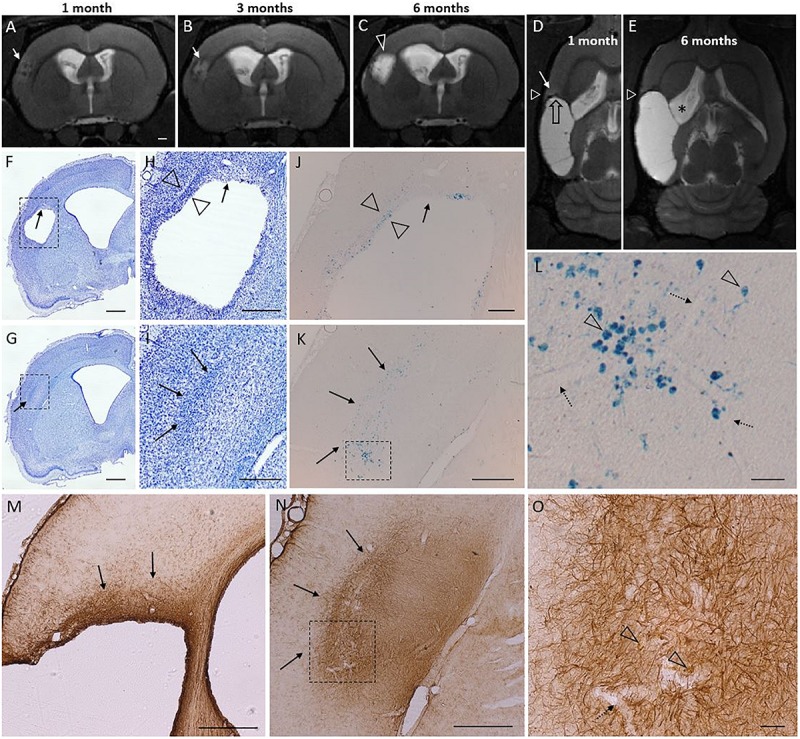
T2 hyperintensity predicts the rostral lesion growth. Lesion continues to expand at its rostral tip which is characterized by glia cloud rather than a tight scar, and both diffuse and phagocyted iron. Iron is partly embedded into the glia scar. Subtle T2 hyperintensity is found to precede the lesion propagation and identify the rostral area to be atrophied in following months. Panel shows atrophy progression in T2-weighted (T2w) MRI in a representative rat from 1 to 6 months post-injury, and histological findings around the rostral tip of the lesion (7 months post-injury) in the same rat. [Two levels of Nissl **(F–I)**, Perl’s Prussian Blue **(J–L)**, and GFAP **(M–O)** stained sections are shown **(F,H,J)** at –0.30 mm, **(M)** at –0.60 mm, and **(G,I,K,L,N,O)** at 0.20 mm from bregma]. **(A–C)** Coronal T2w images captured at the same rostrocaudal level of the same rat at 1, 3, and 6 months post-injury. **(D,E)** Horizontal images from the same rat, allowing elimination of any partial volume effects as a source of the hyperintensity. White triangle indicates the level of coronal images in panels **(A–C)**. Black stripe (T2 hypointensity) along the lesion border was caused by iron residues (black open arrow). Cortical hyperintensity (white arrow in panel **D**) outside the rostral edge of the CSF-filled lesion predicted lesion progression over the following months [compare panels **(D,E)**, rostral growth direction depicted by open black arrow in panel **D**]. Expansion of the cortical lesion was accompanied by the continuous enlargement of the ipsilateral ventricle (asterisk). The hyperintense band was observed in 75% of the TBI animals at 1 month post-injury. By 6 months the hyperintense area had become merged into the lesion cavity. **(F–I)** Nissl and **(M–O)** GFAP stained sections of the same animal show a cloud of astrogliosis, incomplete glial envelope toward the dorso-rostral end of the lesion [arrows in panels **(F,H,M)**], and a thick glial scar next to it [open arrowheads in panel **(H)**]. **(G–I)** Typical to rostral aspect of the lesion was a diffuse glial cloud, extending beyond the immediate lesion edge [arrows in panels **(G–I,M,N)**]. **(J–K)** Prussian blue staining shows the distribution of iron in corresponding locations. **(L)** A higher-magnification prohotomicrograph [dashed box in panel **(K)**] highlights phagocytosed iron (arrowheads), free diffuse iron, and iron associated with blood vessels (dotted arrows). **(O)** Open arrowheads point to iron within the rostral astroglial scar [dashed box in panel **(N)]**. In general, the glia envelope in all cavity cases was tight and narrow in all 3D-directions except rostrally. In the “tightly sealed” directions the lesion expansion seized by 1–3 months. The “leaky rostral end,” however, maintains the hyperintense MR signature of on-going inflammation, and continues to expand. Scale bars: **(A–G)** 1 mm, **(H,I)** 500 μm, **(J)** 200 μm, **(K)** 500 μm, **(L)** 50 μm, **(M,N)** 500 μm, and **(O)** 50 μm.

We observed T2-hyperintensity anterior to the glial scar envelope that surrounded the rostral aspect of the lesion cavity in 75% of the TBI animals at 1–3 months post-injury. By 6 months post-injury, the lesion cavity (i.e., tissue loss) had expanded to cover the entire area of T2 hyperintensity. All lesions with a rostral T2 hyperintensity expanded. Only a single focal case lacked the rostral T2 hyperintensity despite of rostral lesion expansion. Hyperintensity manifested always in the dorso-rostral aspect of the lesion located in the S1 barrel field cortex. The rostral edge of the lesion also showed remarkable iron accumulation visualized in T2w MRI. Figure 7 shows the MRI characteristics of the rostral perilesional cortex and corresponding Nissl, GFAP and Prussian blue stained sections.

### *In vivo* [^18^F]-FEPPA-PET Showed Perilesional Cortex to Be Void of TSPO Overexpression 6 Weeks Post-injury and Thereafter, Suggesting Negligible Levels of Pro-inflammatory Subtype of Reactive Microglia

**FIGURE 8:**
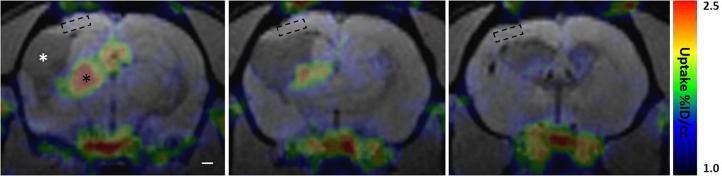
Perilesional cortex and glial envelope show no evident [^18^F]-FEPPA binding indicating lack of translocator protein 18 kDa TSPO overexpression. PET signal color map co-localized with MRI (3D-FISP) at three coronal levels of a representative trauma rat 6 weeks post-injury. [^18^F]-FEPPA-PET shows evident TSPO expression in ipsilateral thalamus (black asterisk) while perilesional cortex shows no evident binding of the tracer. In particular, the location of MRS voxel (dashed box) shows no detectable [^18^F]-FEPPA signal, suggesting absence of reactive pro-inflammatory micro- and astroglia. Resting glia or anti-inflammatory glia does not overexpress TSPO, and thus, is not detected in the PET scan. Cerebrospinal-fluid filled lesion cavity is indicated by white asterisk. Glia envelope around the lesion is also expressing only negligible amounts of TSPO appearing void of any [^18^F]-FEPPA signal. Same animal did show marked [^18^F]-FEPPA signal over the lesion and the perilesional cortex in earlier time point, 2 weeks after the injury, but by 6 weeks the signal is gone (Supplementary Figure 3 shows the evolvement of [^18^F]-FEPPA signal over time along with T2w images). Rostral tip of the lesion (last image) shows no TSPO expression despite on-going growth. Note that the MRI characteristics of the lesion size, lesion surroundings, and overall atrophy are comparable to those shown in Figure 7.

A small cohort of rats with lateral FPI was produced to probe the presence of reactive microglia and astroglia in the perilesional cortex over time by using *in vivo* [^18^F]-FEPPA-PET to map the binding of TSPO radiotracer. TSPO is overexpressed in the mitochondrial membrane of reactive M1 type microglia and reactive astroglia, but not in stable astroglial scar or other types of microglia, including anti-inflammatory M2 type microglia (Beckers et al., 2018). Perilesional cortex showed [^18^F]-FEPPA binding only at early 2-4 wk post-injury time points (Supplementary Figure 3). The cortical signal disappeared by 6 wk post-injury, and remained absent thereafter (Figure 8 and Supplementary Figure 3). In the same images we found massive TSPO signal in the ipsilateral thalamus, reporting on microgliosis (Figure 8 and Supplementary Figure 3). Interestingly, the MRS detected elevated astrocytic marker (Ins) and signs of oxidative stress (GSH) in the [^18^F]-FEPPA-PET negative perilesional cortex. Moreover, the glia scar was devoid of any hot spots of TSPO expression despite the lesion growth was still continuing as verified by MRI follow up.

## Discussion

This study utilized three imaging approaches to reveal chronically on-going cortical posttraumatic inflammation in MRI- perilesional cortex and along the glia scar. Firstly, MRS detected elevated concentrations of inflammation indicating neurochemicals Ins, GSH and GPC+PCh in the MRI- perilesional cortex and, indeed, the pathological levels were sustained 1, 3, and 6 months after head injury. Secondly, T2w MRI a month after injury highlighted on-going inflammation next to the lesion border as hyperintensity which was abolished by 3 months and which preceded, and matched the area of the subsequent lesion growth. Thirdly, *in vivo* [^18^F]-FEPPA-PET showed negligible TSPO binding in the perilesional cortex or along the glia scar 1.5 months post-injury and thereafter suggesting absence of those types of reactive astro- and microglia that overexpress TSPO on their mitochondrial membrane. MRI follow-up also provided a comprehensive view to the progressive atrophy and yielded structural endophenotype classifications. The lesion expansion rate was found to be associated with the maturation stage of the glia scar evaluated by Nissl and GFAP staining. The lesion expansion rate associated only weakly with the dynamics of Ins, GSH and GPC+PCh in the preserved cortex.

### Spectroscopic Inflammation Markers Were Elevated Throughout the 6 Months Follow-Up in the MRI Negative Perilesional Cortex

Spectroscopy voxel of 1 mm × 3 mm × 5 mm contained S1BF, dysgranular zone (S1DZ), front limb region (S1FL) (minor portion of hindlimb region S1HL), ending caudally in parietal association (PtA) and secondary visual (V2L) cortical regions. Lesion covered large portions of the primary and secondary somatosensory cortices (S1 and S2), primary auditory cortex (Au1), dorsal and ventral areas of the secondary auditory cortex (AuD and AuV), dorsal and rostral areas of posterior parietal cortex (PtPR and PtPR) extending often to ectorhinal cortex (Ect), perirhinal cortex (PRh), and entorhinal cortex (Ent) according to Paxinos rat atlas (Paxinos and Watson, 1998). Spectroscopy voxel avoided the lesion and probed the MRI- cortex revealing chronically elevated concentrations of inflammatory markers Ins, GSH and GPC+PCho. Thus, MRS revealed sustained chronic inflammatory-stage undetected by structural MRI.

### Methodological Considerations – MRS of Atrophied Cortex

The target of the MRS measurement was the healthy appearing perilesional cortex, which has been largely overlooked in previous MRS studies. Lescot et al. (2010) focused their localized MRS study over the primary contusion in the cortex and excluded subcortical structures, but described only the first 7 days after lateral FPI. Harris et al. (2012) comprehensively described the changes in the entire neurochemical profile 2 weeks after the TBI in CCI rat model with localized 1H-MRS, but the horizontally localized voxel missed a major portion of the cortical areas we are covering, and included both the primary lesion and the subcortical structures that we are avoiding.

While assessing the neurochemical changes over time in TBI animals with progressive atrophy, it is crucial that the contributions of CSF volume fraction, and white matter into the cortical MRS voxel are accounted for in the analysis. We report here the absolute concentrations obtained from LCmodel analysis corrected for the CSF volume fraction and for the 99% water content of CSF with tissue water content approximated to 80%. Additional spectra obtained from a voxel located entirely within the lesion cavity showed that no Ins, GSH or choline signal arose from the CSF-filled-cavity (data not shown). In Supplementary Material we report the concentrations relative to total creatine, which in principle normalizes the results to the total cell count, and thereby takes into account the partial atrophy. Creatine levels, however, are susceptible for changes due to the pathology. Taken together, both the absolute concentrations and concentrations normalized to total creatine yield the same main results.

Analysis of the dynamic changes between 1 and 3 months were also repeated with regression analysis including white matter (WM) fraction as covariate in repeated measures ANCOVA to ensure that the observed changes were not due to the changes in voxel WM content. WM has higher Ins content than gray matter. WM content was obtained by manually outlining the portion of external capsule that overlapped the MRS voxel volume. The results were found to be independent of the WM volume fraction.

Aging effect was taken into account by adding a group of naïve 8.5 months old rats to the study. They were age-matched control for the 6 months post-injury time point and thus depicted the level of Ins in healthy, aged cortex. It has been shown that the aging lead to elevated astroglia markers on the cortex (Harris et al., 2014, 2015).

### Chronically Elevated Myo-Inositol May Reflect Cortical Plasticity Beyond the Gliosis

Myo-inositol is a marker of all glia: both the reactive gliosis or the stable astroglia scar. It has been recognized as a marker for inflammation (Harris et al., 2015) but it has also multiple other roles (Duarte et al., 2012). Ins concentrations have been positively correlated with GFAP and S100b positive cell densities in hippocampus of injured rats in other preclinical brain insult models (Filibian et al., 2012; Pascente et al., 2016). It is also found to be elevated in CCI model in the primary injury site 14 days post-injury (Harris et al., 2012) and in lateral FPI model in ipsilateral hippocampus at 5 months post-LFPI (Immonen et al., 2009b). We found elevated concentrations of Ins in all TBI animals at 1 month post-injury and group average remained elevated from 1 to 6 months post-injury despite dual dynamics: “attenuating” or “on-going.” The MRS voxel avoided the areas of dense GFAP staining close to the lesion border or along subcortical WM. Importantly, in the context of chronic perilesional cortex that undergoes many forms of plastic remodeling as a delayed response to the injury, the increases in Ins concentration may also associate with the demand for synthesis of inositol-containing phospholipids during synaptogenesis, axonal growth and myelination, as has been suggested in developing brain (Tkac et al., 2003; Kulak et al., 2010; Duarte et al., 2012).

### Delayed Increase in Total Choline Suggests Higher Membrane Turnover 6 Months Post-injury Than 1 Month Post-injury

Increase in total choline (GPC+PCho) is an indicator of membrane turnover (degradation or proliferation) and its elevation in white matter has been used as a marker of diffuse axonal injury and inflammation (Holshouser et al., 2005; Babikian et al., 2006; Shutter et al., 2006; Govind et al., 2010). Choline has been found to be elevated at the primary injury site and surrounding tissue 14 days post-CCI (Harris et al., 2012). Interestingly, our data shows that GPC+PCho in healthy appearing perilesional cortex is not elevated yet at 1 month but becomes elevated 3–6 months post-injury, while Ins is elevated already at 1 month time point. This suggests choline elevation to be associated with membrane turnover processes unrelated to gliosis. These exactly same cortical areas have been shown to undergo number of degradative and regenerative plastic changes in vasculature, extracellular matrix, cellular content, GABAergic interneurons, axonal integrity and connectivity, myelin content, etc. (Avramescu and Timofeev, 2008; Hayward et al., 2010; Cantu et al., 2015; Lehto et al., 2017; Ndode-Ekane et al., 2018; Pitkanen et al., 2018). However, these phenomena should already show at 1 month MRS, while the late increase of choline suggests more delayed processes to take place. Chronically the cortex gets thinner and “squeezed,” thus apparently loses some of its extracellular space. While being a complicating factor in the analysis of the absolute concentrations, this phenomenon may also be reflected in the total choline concentration.

### High Levels of Antioxidant Glutathione Suggest Sustained Oxidative Stress 6 Months Post-injury

Glutathione (GSH) is an antioxidant secreted by astrocytes to battle the reactive oxygen species produced by neurons under inflammatory conditions. We found elevated GSH levels in the ipsilateral cortex 1–6 months after TBI while glia marker Ins levels were elevated as well. Increase in GSH levels has been reported in injured rat hippocampus during epileptogenesis after status epilepticus (Filibian et al., 2012) and in cingulate of mild cognitive impairment patients (Duffy et al., 2014). Treatments against oxidative stress have been recently demonstrated to alleviate several disorders in preclinical studies of different brain insults (Pauletti et al., 2017; Frigerio et al., 2018). On the contrary, cortical GSH has been found to be depleted in patients with brain disorders, such as Alzheimer’s, mild cognitive impairment, multiple sclerosis, repeated concussions in retired rugby players, as well as upon aging (Benzi et al., 1989; Geremia et al., 1990; Choi et al., 2011; Emir et al., 2011; Mandal et al., 2015; Chiang et al., 2017; Gardner et al., 2017). Also iron-mediated inflammation causes depletion of GSH. Decreased levels of blood glutathione peroxidase in chronic phase after severe TBI has been associated with greater disability (Licastro et al., 2016). In our study, the GSH elevation in perilesional cortex months after injury indicates that the brain’s ability to combat oxidative stress is increased. GSH levels may increase when astrocytes are chronically activated and recruited, and we observed sustained high concentrations of both Ins and GSH in chronic TBI animals. This suggests chronically sustained, elevated perilesional astrocytic activity, compensatory increase of GSH production due to the sustained battle against oxidative stress, or low GSH depletion. Elevated antioxidant GSH concentration may also associate with the fact that the surviving cortical tissue has been shown to exhibit spontaneous epileptiform activity in a subset of animals in FPI (Reid et al., 2016) and CCI (Yang et al., 2010) models.

### [^18^F]-FEPPA-PET Binds to TSPO Overexpressing Reactive Microglia in Thalamus, but Shows No Prominent Binding in Cortex After 1.5 Month Post-injury

Translocator protein 18 kDa (TSPO), also known as peripheral benzodiazepine receptor (PBR), targeting radiotracers are the largest group of PET tracers for neuroinflammation, and [^18^F]-FEPPA is one of the new generation TSPO tracers with improved binding efficacy (Wilson et al., 2008; Vignal et al., 2018). In a supplementary group of animals (*N* = 3) we saw clear TSPO expression in contusion site at acute stage post-injury 2–4 weeks post-injury, while the cortical [^18^F]-FEPPA signal was abolished by 6–8 weeks post-injury PET scans in the same animals. This is in line with experimental TBI studies utilizing TSPO-PET tracers that have shown the expression to peak 6–7 days post-injury in the injured cortex due to the reactive microgliosis response, stay elevated at 10 days due to reactive astroglia at and around the contusion, and decay by 14–28 days post-injury (Yu et al., 2010; Wang et al., 2014; Israel et al., 2016; Missault et al., 2018). Subacute TSPO expression along the edges of the CCI lesion was recently shown to be elevated 7 days post-injury and returning close to the control level by day 21 post-injury. Interestingly however, the TSPO expression along the lesion edges at day 21 correlated with the long-term outcome in pentylenetetrazol-evoked seizure susceptibility and anxiety in open field test (Missault et al., 2018). Only one study utilizing [^18^F]-FE-DAA1106 after lateral FPI has reported *in vivo* observable signal up to 9 weeks post-injury in the injured cortex. In the same study, the autoradiography demonstrated TSPO binding along subcortical white matter and along the edges of the cortical lesion to be detectable up to 3 months post-injury (Yu et al., 2010). At this late stage the origin of the signal were a subset of GFAP expressing cells (astroglia) and not the Iba1 positive (microglia) cells (Yu et al., 2010; Wang et al., 2014). Importantly, the perilesional cortex distant from the immediate lesion edge appeared free of TSPO overexpression. [^18^F]-FEPPA PET is highly sensitive in detecting pro-inflammatory reactive microgliosis, and in line with literature, we observe strong binding in injured thalamus. Sustained thalamic inflammation and associated reactive gliosis is well documented in this animal model (Pierce et al., 1998; Lehto et al., 2012), and in human TBI (Papadopoulos and Lecanu, 2009; Ramlackhansingh et al., 2011; Coughlin et al., 2015; Faden et al., 2016). However, it is broadly recognized that the TSPO tracers have their limitations as inflammation markers: TSPO is not overexpressed in all subtypes of reactive glia, nor is it expressed in a “stable” astroglia scar (Virdee et al., 2012; Wu et al., 2013; Amhaoul et al., 2014; Tronel et al., 2017). Recent intriguing findings of Beckers et al. (2018), demonstrate that TSPO expression depends on the microglia polarization. TSPO expression was selectively increased in pro-inflammatory (classically activated M1 type) microglia but not in anti-inflammatory (alternatively activated M2 type) microglia, with activation evoked by IL-4 injection (Beckers et al., 2018). Thus, our preliminary observations of perilesional cortex being TSPO-negative do not suggest that the reactive micro-or astroglia would be absent in chronic stage post-injury, since the presence of them is well documented (Ndode-Ekane et al., 2018) but rather that their activation state has shifted to anti-inflammatory (M2). However, many factors of the TSPO expression in different disease conditions are still unknown.

[^18^F]-FEPPA-negativity of the perilesional cortex indicates that the elevated myo-inositol levels in MRS do not originate from the TSPO expressing glia types, but others. Therefore, we speculate that the mismatch between myo-inositol and TSPO (“*Ins-TSPO mismatch*” i.e., myo-inositol concentrations being elevated but TSPO-PET being negative) could potentially identify the areas of M2 type glial activity, e.g., on-going plasticity or on-going glia scar built up of post-injury cortex. Furthermore, the results suggest that the cause for elevated antioxidant GSH levels 3–6 months after TBI are not the reactive oxygen species resulting from local M1 type of microglial response.

### Structural Endophenotypes, With Characteristic Speed of Cavity Formation and Glia Scar Maturation, Reflect the Efficacy of the Immunodefence

The existence of different structural endophenotypes (focal and cavity forming) of lateral FPI rats is a fact that we have been observing in several of our cohorts, and obviously the spectrum of the different lesion types spans the sizes between these two ends at some point of the lesion expansion. Here, we determined the endophenotypes at 1 month post-injury, corresponding a clinically relevant time point for delayed therapies, when the differences in the lesion progression rate can already be seen. Structural endophenotyping is informative as itself. In cavity forming endopenotype the heavy inflammatory response occurs fast, and tight, thin glia scar is formed, while in the focal-cases the response is slower or less efficient and glia scar is thick and diffuse. This suggests that the speed of cavity formation may serve as one indicator of immunodefence efficacy. Moreover, the fact that the proportional growth between 1 and 3 months is largely seized in cavity forming cases while is still 115% in focal cases and 26% in intermediate cases may have major impact in case-selection of neuroprotective trials.

### On-Going Inflammation and Long Lasting Lesion Growth in the Rostral Tip of the Lesion

The systematic observation throughout the TBI cohort was that in the chronic phase the dorso-rostral edge of the cortical lesion was the site of on-going lesion expansion, immature glia scar and on-going inflammation. In MRI the rostral lesion end was always associated with hematomas, iron residues and often with the T2w signature of on-going inflammation. In endpoint histology the glia envelope of the lesion cavity was wide and disperse at this rostral location while fulfilling the characteristics of and mature, tight glia scar elsewhere. This could indicate that the rostral perilesional cortex is more vulnerable, fails to create the glia shield or have sustained plasticity that hinder the glia envelope formation. The dorsal perilesional tissue also suffers from severe hypoperfusion as well as vascular abnormalities (Hayward et al., 2010), which may predispose it to any further stressors or prevent it from forming the mature glia scar. Certainly, the results imply that the cortical tissue rostrally is not in a stable state, but dealing with infiltrating leukocytes and related stress factors month after month, possibly leading to a local dysfunction. Interestingly, this particular location has also been indicated as putative seizure onset zone in lateral FPI rat model of posttraumatic epilepsy (Reid et al., 2016).

## Conclusion

There is a need for *in vivo* imaging approaches that could assess the putatively sustained or chronically evoked cortical inflammation after TBI. Current scientific consensus is that central nervous system inflammation is one factor determining the long-term outcome and that it offers a promising target for therapies – even late onset treatments. Both the pro-inflammatory and anti-inflammatory glial activity have disease modifying effects, and discerning the two is a major challenge. Lateral FPI rat model is widely utilized in pre-clinical drug research, and identifying the animals suffering from chronically on-going cortical inflammation will aid to both enrich the population in pre-clinical trials as well as tailor optimally timed therapies. Robust, fast T2w acquisitions are suitable for screening purposes since they are rich in structural information and detect the types of inflammation that attract edema. Evaluating the maturation stage of the glia envelope of the lesion by its T2w MRI properties sheds light in to the disease mechanisms and the sustained plasticity in the lesion edges. Yet, subtle neuroinflammation remains undetected by structural MRI. We found inflammatory MRS profile of myo-inositol, glutathione and choline to be sensitive enough to detect inflammation in MRI-negative cortex. The dynamics of the MRS markers did not go hand-in-hand with the lesion growth rate suggesting independent inflammatory processes along the lesion edge versus further in the cortex. Observations with the third imaging approach, [^18^F]-FEPPA-PET, suggest that TSPO overexpression, that occurs in pro-inflammatory subtype of reactive microglia, may not be present in chronic posttraumatic cortex. Instead, the follow-up of the TSPO expression might serve in indicating the shift in microglial polarization from pro-inflammatory to anti-inflammatory state in injured cortex. This is crucial information when planning the imaging paradigm and timing of anti-inflammatory treatment trials. Taken together, when probing different aspects of posttraumatic inflammatory processes the MRI, MRS, and PET clearly complement each other.

## Data Availability

The datasets generated for this study are available on request to the corresponding author.

## Ethics Statement

All animal procedures were approved by the Animal Care and use Committee of the University of Eastern Finland and performed in accordance with the guidelines of European Community Council Directives 2010/63/EU and 86/609/EEC.

## Author Contributions

RI, OG, and AP conceived and designed the study. OG and AP provided the resources and research facilities. AY and RI collected and analyzed the MRI and MRS data, and analyzed the histology. PP produced the PET radioligand. KJ conducted the PET measurements. AY, RI, and AP wrote the manuscript. KJ, PP, and OG wrote the sections of the manuscript. All authors revised the manuscript, and read and approved the submitted version.

## Conflict of Interest Statement

The authors declare that the research was conducted in the absence of any commercial or financial relationships that could be construed as a potential conflict of interest.

## Footnotes

1 http://aedes.uef.fi/
