# Supplementary material for: MRS Reveals Chronic Inflammation in T2w MRI-Negative Perilesional Cortex – A 6-Months Multimodal Imaging Follow-Up Study
Source: Front Neurosci. 2019 Aug 16;13:863. doi: 10.3389/fnins.2019.00863 (PMC6707062; doi:10.3389/fnins.2019.00863)
Supplement: Supplementary file 1 [file Data_Sheet_1.PDF]

SUPPLEMENTARY DATA

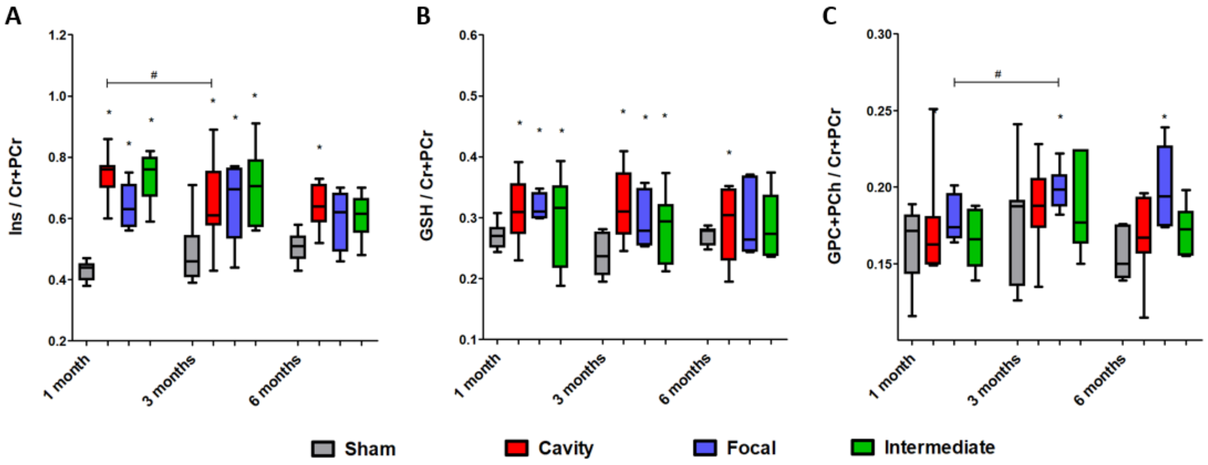

Supplementary Figure 1. Concentrations of the MRS inflammatory markers normalized to creatine (Cr+PCr) concentration, and their dynamics over time in each structural endophenotype. Changes in myoinositol (Ins), glutathione (GSH) and total choline (GPH+PCho) normalized to creatine concentration are yielding the same main findings than obtained with absolute concentrations (Fig 5D-F). However, Ins/Cr+PCr shows decrease in cavity group from 1 month to 3 months while remaining high in focal and intermediate groups (A). This suggests earlier attenuation of inflammation in cavity forming endophenotype. Also, choline compounds show significant increase of GPC+PCho/Cr+PCr between 1 and 3 months in focal group, while the same effect remains as a trend in other groups (C). This suggests accelerated and chronically sustained membrane turnover in focal group.

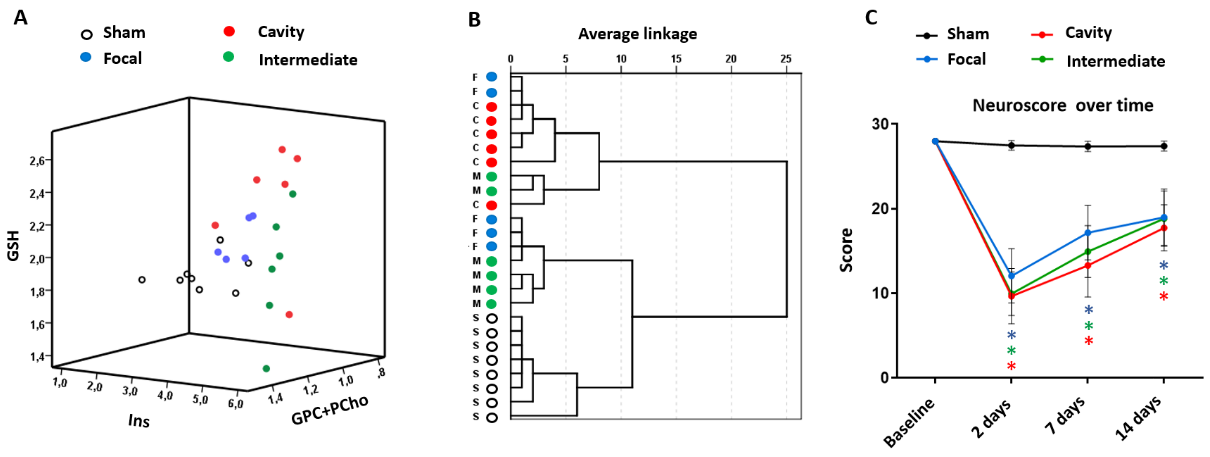

Supplementary Figure 2. Combined information of the three MRS inflammatory markers results in non-complete clustering of the structural endophenotypes. (A) 3D scatter plot of myoinositol (Ins), glutathione (GSH) and total choline (GPC+PCho) at 1 month post-injury. **(B)** Dendrogram shows the result of the unsupervised hierarchical clustering including the data of Ins, GSH and GPC+PCh 1 month post-injury. Clustering separates the shams, but fails in differentiating the endophenotypes: 2/5 focal cases are falsely clustered together with cavity cases, and intermediate cases are divided into both cavity and focal clusters. The unsupervised hierarchical clustering was done using average linkage between groups with square root Euclidean distance. **(C)** Motor recovery in composite neuroscore test during the first 14 days post-injury did not differ between endophenotypes. Deficits in postinjury motor function were significant in all endophenotypes as compared to shams (*, $p<0.05$). Neuroscore results between groups were compared using Kruskall-Wallis for day 2 and day 14, and for the motor recovery from day 2 to day 14, $p<0.05$ was considered significant. The motor recovery was calculated as a recovery of neuroscore points from day 2 to day 14 normalized to the magnitude of the drop at day 2 [(NS(14d)-NS(2d))/(NS(baseline)-NS(2d))].

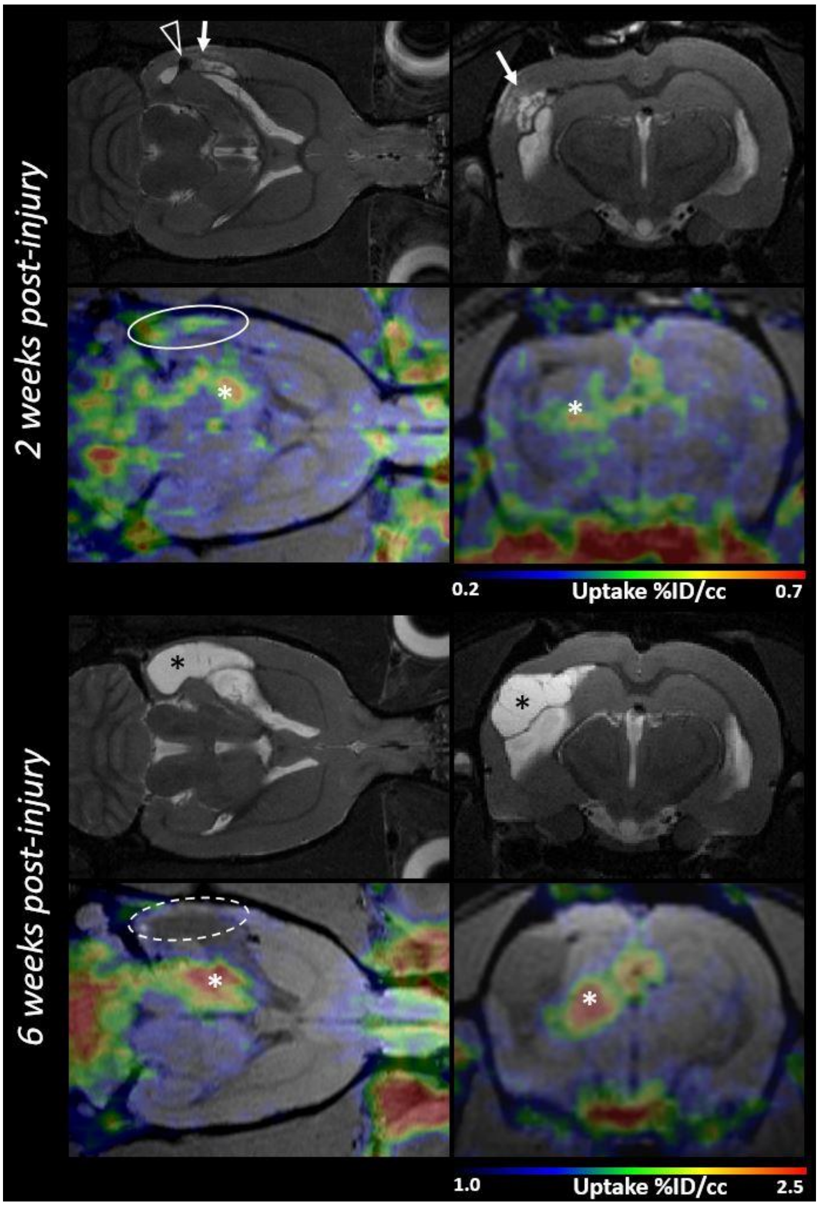

Supplementary Figure 3. [^{18}F]-FEPPA-PET detects cortical inflammation at 2 weeks postinjury but no longer at 6 weeks postinjury. [^{18}F]-FEPPA-PET signal (color) overlaid on T1-weighted FISP 3D images and corresponding T2-weighted (T2w) MRI of the same animal are shown in horizontal and coronal planes over time (2 weeks time point on top, 6 weeks on the bottom). Upon the atrophy progression from 2 weeks to 6 weeks post-injury the [^{18}F]-FEPPA signal around the cortical lesion gets weaker or absent (solid circle at 2 weeks time point with evident TSPO tracer signal, dashed circle at 6 weeks with negligible signal). T2w images show how the early hemorrhagic (arrowhead) and edematic (arrow) lesion becomes cavity (asterisk) enclosed by glia envelope by the 6 weeks scan. There was no apparent TSPO expression along glia scar 6 weeks post-injury or later (later data not shown). Nor was there apparent TSPO expression in the perilesional cortex 6 weeks post-injury or later. Simultaneously, through all time points (2 weeks – 1 year post-injury) the thalamic TSPO expression was evident (white asterisk) in all time points. Cerebellar and olfactory signal depicts the normal biodistribution of TSPO expression. T2w MRI images obtained at day 9 post-injury are shown as reference to 2-week PET and those obtained 7 weeks post-injury are shown as reference to 6-week PET. Part of the radiotracer injected for 2-week scan remained in the tail causing the delivered dose as %ID/cc to be smaller than at 6-weeks.
